# Molecular Determinants of Regulatory T Cell Development: The Essential Roles of Epigenetic Changes

**DOI:** 10.3389/fimmu.2013.00106

**Published:** 2013-05-10

**Authors:** Yohko Kitagawa, Naganari Ohkura, Shimon Sakaguchi

**Affiliations:** ^1^Department of Experimental Immunology, World Premier International Immunology Frontier Research Center, Osaka UniversitySuita, Japan; ^2^Department of Experimental Pathology, Institute for Frontier Medical Sciences, Kyoto UniversityKyoto, Japan

**Keywords:** regulatory T cells, Foxp3, epigenetics, DNA methylation, adaptability, plasticity, lineage specification

## Abstract

Regulatory T (Treg) cells constitute a distinct T cell subset, which plays a key role in immune tolerance and homeostasis. The transcription factor Foxp3 controls a substantial part of Treg cell development and function. Yet its expression alone is insufficient for conferring developmental and functional characteristics of Treg cells. There is accumulating evidence that concurrent induction of Treg-specific epigenetic changes and Foxp3 expression is crucial for lineage specification and functional stability of Treg cells. This review discusses recent progress in our understanding of molecular features of Treg cells, in particular, the molecular basis of how a population of developing T cells is driven to the Treg cell lineage and how its function is stably maintained.

## Introduction

Regulatory T (Treg) cells represent a subset of CD4^+^ T cells specialized for the maintenance of immune tolerance and homeostasis by suppressing excessive and aberrant immune reactions harmful to the host. While the majority of Treg cells develop in the thymus, some are induced from naïve CD4^+^ T cells in the periphery. In order for Treg cells to exert their regulatory functions, constitutive expression of the transcription factor Foxp3 is essential (Hori et al., 2003; Williams and Rudensky, 2007). The pivotal roles of Foxp3 in Treg cell function and development are best illustrated by the manifestation of multi-organ autoimmune inflammation in FOXP3-deficient Immunodysregulation Polyendocrinopathy Enteropathy X-linked syndrome (IPEX) patients and Scurfy mice (Bennett et al., 2001; Brunkow et al., 2001; Fontenot et al., 2003). Also as demonstrated by retroviral transduction of Foxp3 in conventional CD4^+^ T (Tconv) cells, Foxp3 expression, combined with T cell receptor (TCR) stimulation, enables the acquisition of Treg properties including suppressive function, hyporesponsiveness to TCR stimulation, and up-regulation of Treg-associated molecules including CTLA-4, GITR, and CD25 (Hori et al., 2003; Yagi et al., 2004). Foxp3 is therefore recognized as a master regulator of Treg cell function and development.

In addition to the expression of Foxp3, several comprehensive analyses have recently revealed possible involvement of other molecular mechanisms in the development of Treg cells. For example, genome-wide comparison of DNA methylation status in Tconv and Treg cells has demonstrated the presence of Treg-specific DNA hypomethylation in the genes associated with Treg function (Schmidl et al., 2009; Ohkura et al., 2012). Proteomic analysis in Treg cells indicates that Foxp3 forms complexes with a number of co-factors to exert cooperative effects upon interaction (Rudra et al., 2012). Furthermore, combinations of Foxp3 with several other transcription factors are able to induce a common Treg-type gene expression pattern, which cannot be achieved solely by Foxp3 (Fu et al., 2012). These findings suggest that the generation of functional Treg cells requires more than just the expression of Foxp3.

With the indispensable roles of Foxp3 in exerting Treg cell function, stable expression of Foxp3 is a critical factor in Treg cell development. However, from fate-mapping studies using Foxp3 reporter mice, it is becoming apparent that while the majority of Treg cells are stable, a minor fraction of Foxp3^+^ T cells shows plasticity and becomes non-Treg cells by losing Foxp3 (Komatsu et al., 2009; Zhou et al., 2009). Furthermore, both human and murine naïve CD4^+^ T cells transiently express Foxp3, without acquiring suppressive function (Allan et al., 2007; Wang et al., 2007; Miyao et al., 2012). These observations suggest the existence of two types of Foxp3^+^ T cells, stable functional Treg cells and Foxp3^+^ naïve-like non-Treg cells, and raise questions regarding the mode of action of Foxp3 in these two populations. Although both populations express Foxp3, Foxp3^+^ naïve-like non-Treg cells lack a significant part of Treg-specific molecular features such as epigenetic modifications. These findings prompt us to reconsider the molecular mechanisms underlying Treg cell development. In this review, we discuss key molecular features that make up functional Treg cells.

## CD4^+^Foxp3^+^ T Cells are Not Always Treg Cells

In most physiological settings, CD4^+^Foxp3^+^ T cells stably maintain suppressive functions irrespective of environmental changes. However, recent studies suggest that the link between Foxp3 expression and suppressive activity is not so clear-cut, as there are a number of anomalies for this molecular definition of Treg cells. One example is a fraction of human Foxp3^+^ T cells. CD4^+^Foxp3^+^ T cells in humans can be divided into three subgroups; CD45RA^+^ Foxp3^lo^ naïve Treg cells, CD45RA^−^ Foxp3^hi^ effector Treg cells, and CD45RA^−^ Foxp3^lo^ T cells, and the last does not possess suppressive function despite the expression of Foxp3 (Miyara et al., 2009). In line with this, human naïve T cells express Foxp3 upon TCR stimulation, yet this Foxp3 expression is transient and does not confer suppressive property (Wang et al., 2007). Similarly, CD4^+^Foxp3^lo^ T cells are observed as a minor fraction of activated Tconv cells in mice; these cells lack Treg-type gene expression and suppressive activity and their unstable expression of Foxp3 results in the generation of exFoxp3 T cells capable of producing inflammatory cytokines (Miyao et al., 2012). These findings indicate that Foxp3 is not exclusively expressed in Treg cells.

Consistently, Foxp3 expression can be induced by some transcription factors, irrespective of whether it accompanies Treg function or not. There are a number of molecules identified to initiate and/or enhance the transcription of Foxp3, such as Smad3, NFAT, Nr4a2, and AP-1 (Mantel et al., 2006; Tone et al., 2008; Sekiya et al., 2011). This indicates that the combination of signals activating these molecules is sufficient to induce Foxp3 expression. In fact, in response to TCR stimulation and TGF-β signaling, a substantial proportion of naïve CD4^+^Foxp3^−^ T cells express Foxp3. However, murine *in vitro*-induced Treg (iTreg) cells have been revealed to differ from thymus-derived Treg (tTreg) or periphery-derived Treg (pTreg) cells *in vivo*. Firstly, they have only partial coverage of Treg-type gene expression profile (Sugimoto et al., 2006; Hill et al., 2007). Secondly, when antigen-specific iTreg cells are transferred into normal mice and immunized with the specific antigen, Foxp3 expression is rapidly lost (Chen et al., 2011). Furthermore, while *in vivo*-generated Treg cells are able to prevent colitis development following transfer of CD4^+^CD45RB^high^ T cells into lymphopenic mice, the same number of iTreg cells can only moderately suppress the disease progress, partially due to the gradual loss of Treg signature molecule expression (Ohkura et al., 2012). In addition, human naïve T cells also express FOXP3 upon TCR and TGF-β stimulation, yet these iTreg cells are reported to lack suppressive function and produce pro-inflammatory cytokines (Tran et al., 2007). Therefore, *in vitro* generated iTreg cells are another example of Foxp3^+^ naïve-like non-Treg cells. As these findings demonstrate, activating Foxp3 transcription does not necessarily indicate the generation of Treg cells, suggesting the importance of widening our focus onto other elements required for Treg cell development and function.

## The Molecular Basis of Foxp3-Dependent Treg Characteristics

Foxp3 expression does not always correlate with Treg function. In addition, at the molecular level, the contribution of Foxp3 to the Treg-specific gene expression appears to be limited (46% of upregulated genes and 28% of downregulated genes in natural Treg cells were Foxp3-dependent) (Hill et al., 2007). This notion is supported by the analysis of Foxp3-binding sites in Treg cells; only a small proportion of the genes differentially expressed in Treg cells are bound and directly regulated by Foxp3 (Zheng et al., 2007). Collectively, these findings suggest that Foxp3 is an essential factor for modulating a substantial part of Treg cell properties, yet Foxp3 alone is insufficient to convert non-Treg cells into Treg cells with full Treg-type gene expression and function. Given the major loss of Treg cell function upon deletion of Foxp3, it is likely that the mode of action of Foxp3 is different in functional Treg cells and Foxp3^+^ naïve-like non-Treg cells.

**Figure 1:**
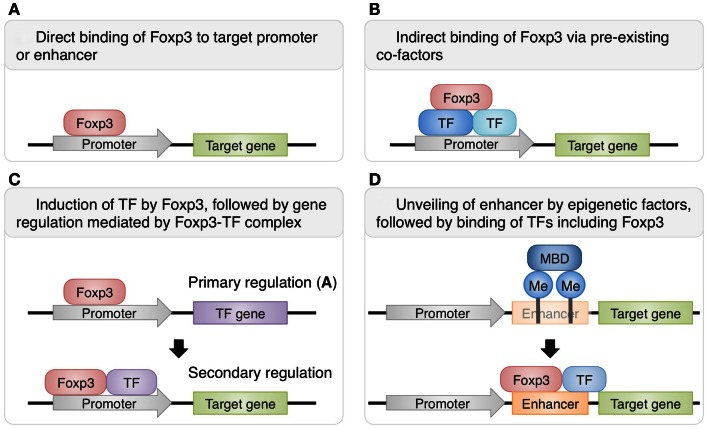
**Various mechanisms of Foxp3-dependent gene regulation in Treg cells**. Some genes are directly regulated by Foxp3 alone **(A)**, while others require the protein complexes containing Foxp3 and its co-factors for transcriptional regulation. Foxp3 can interact with pre-existing transcription factors such as Runx1 and Ets-1 **(B)** or with direct targets of Foxp3-mediated gene regulation, such as GATA-3 **(C)** (Rudra et al., 2012). Furthermore, there are also genes regulated by both Foxp3 and epigenetic changes. For example, at *Foxp3* locus, epigenetic modifications unveil normally hidden enhancer and allow the transcriptional activation by Foxp3 and its co-factors **(D)** (Floess et al., 2007; Schmidl et al., 2009; Zheng et al., 2010).

There are several known mechanisms of Foxp3-mediated transcriptional control (Figure 1). While some gene expression in Treg cells is directly modulated by the binding of Foxp3 to their promoters or enhancers, other gene expression requires interaction of Foxp3 with other transcription factors. Recently, Rudra et al. (2012) identified the comprehensive list of proteins forming complexes with Foxp3 in Treg cells and revealed that a number of these co-factors are transcription factors directly upregulated by Foxp3, suggesting that direct up-regulation of co-factors by Foxp3 is followed by secondary regulation of gene expression by the complexes of Foxp3 and its co-factors. In fact, it has been shown that interactions of Foxp3 with Runx1/Cbfβ, NFAT, or Gata-3 are crucial for the Foxp3-dependent gene expression and consequently Treg cell function (Wu et al., 2006; Ono et al., 2007; Kitoh et al., 2009; Rudra et al., 2012). Another recent study has shown that co-expression of Foxp3 with at least one of the “quintet factors” which include five transcription factors GATA-1, IRF4, Lef1, Ikzf4, and Satb1 induces the same pattern of gene expression covering a substantial part of Treg signatures, which is not achieved by the expression of Foxp3 alone (Fu et al., 2012). Therefore, transcriptional regulation by Foxp3 can be direct or indirect, and the latter involves recruitment of co-factors to expand and specify Foxp3 targets. The composition of Foxp3-containig complexes is likely to be variable at different genomic loci and may also be influenced at the cellular level by immunological contexts, allowing dynamic regulation of Foxp3-dependent transcription programs.

In this regard, Foxp3 exerts significant impact on the phenotypes and function of Treg cells by cooperating with other transcriptional factors. Foxp3^+^ naïve -like non-Treg cells observed in both humans and mice lack the expression of the majority of Treg-associated molecules (Miyara et al., 2009; Miyao et al., 2012), and this may be partially attributed to the lack of Foxp3 interaction with co-factors and consequently the lack of Treg phenotypes and function. As illustrated by iTreg cells induced *in vitro*, Foxp3 expression can be induced by activating a set of transcription factors and altering histone modifications at promoter and enhancer regions. However, for the development of functionally stable Treg cells, it is likely to require Foxp3 expression, together with the expression of its partner molecules, and also other factors regulating Foxp3-independent features of Treg cells.

## Epigenetic Features of Treg Cells

The heterogeneity of CD4^+^Foxp3^+^ T cells shows the need for an additional marker in order to distinguish between functional Treg cells and Foxp3^+^ naïve-like non-Treg cells. One of key differences between these two populations is the stability of Treg phenotypes. In search of the molecular determinant of this feature, recent studies have focused on the epigenetics, a more stable level of transcriptional regulation. Epigenetic changes include histone modification, DNA methylation of CpG residues, and nucleosome repositioning. These events alter the accessibility of transcription factors and RNA polymerase to regulatory regions of the genome, thereby stably switching on and off the gene transcription. This level of transcriptional regulation is particularly important in cell differentiation in eukaryotes, allowing the stability of cell type-specific gene expression.

Several groups have discovered that such epigenetic changes take place in the course of Treg cell differentiation. Treg cells are associated with DNA hypomethylation at *Foxp3* conserved non-coding sequence 2 (CNS2) and it was shown to be required for stable expression of Foxp3 (Floess et al., 2007; Kim and Leonard, 2007). Furthermore, DNA demethylation also concurrently takes place within the genes known as “Treg signatures,” namely *Foxp3*, *Ctal4*, *Ikzf2* (Helios), *Ikzf4* (Eos), and *Tnfrsf18* (GITR) (Ohkura et al., 2012). These changes are specific to Treg cell development and not induced in response to TCR or TGF-β stimulation (Polansky et al., 2008; Ohkura et al., 2012). Accordingly, *in vitro* generated iTreg cells and Foxp3^+^ naïve-like non-Treg cells observed in humans and mice show the lack of Treg-specific DNA hypomethylation, which correlates with the lack of a significant part of Treg-type gene expression and stability of Treg signature molecule expression (Miyara et al., 2009; Miyao et al., 2012; Ohkura et al., 2012). In addition to stabilizing Treg phenotypes, epigenetic components of Treg cells also appear to regulate the Treg-type gene expression pattern, either independently of Foxp3 or cooperatively with Foxp3. Gene expression analysis of *Foxp3*-null Treg cells, which contain disrupted *Foxp3* gene and fluorescent marker controlled by the *Foxp3* promoter, shows that a set of genes including many of the Treg signatures are expressed even without Foxp3 expression and that Foxp3 amplifies the pre-established gene expression profile (Gavin et al., 2007). These *Foxp3*-null Treg cells also possess Treg-specific DNA methylation pattern, which correlates with the corresponding gene expression (Ohkura et al., 2012).

**Figure 2:**
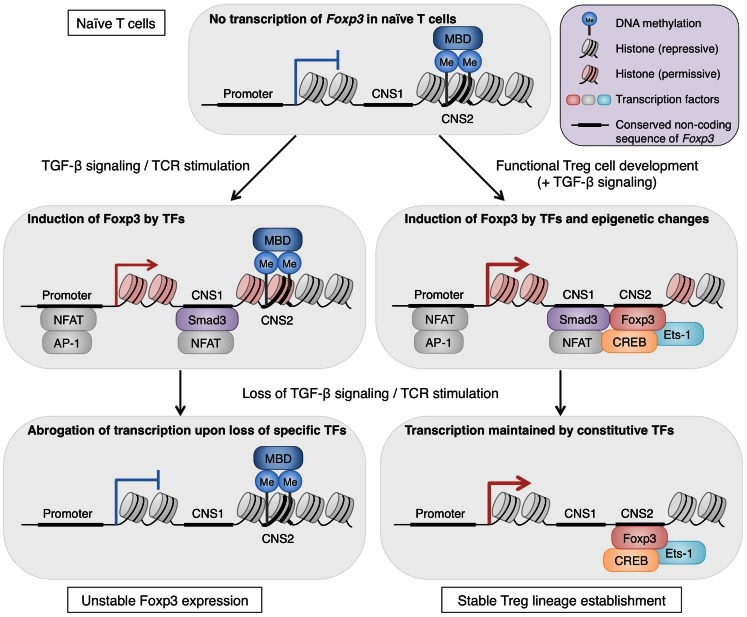
**The roles of epigenetic changes in stabilizing *Foxp3* expression**. Epigenetic changes during Treg cell development are important for long-term stability of Treg phenotypes, particularly Foxp3 expression. *Foxp3* CNS2 in naïve CD4^+^ T cells shows repressive histone markers, low accessibility for transcription factors, and methylated CpG residues, likely attracting methyl-CpG-binding domains (MBDs). In the course of Treg cell development, epigenetic changes take place and accessibility of CNS2 increases by DNA demethylation, histone modifications, and possibly nucleosome repositioning (Ohkura et al., 2012; Samstein et al., 2012). The CNS2 region serves as an enhancer for *Foxp3* transcription and is bound by transcription factors such as Foxp3, Ets-1, and CREB. These epigenetic alterations are maintained irrespective of environmental changes and thus allow stable *Foxp3* transcription by constitutively expressed transcription factors. In contrast, Foxp3 expression induced by TGF-β signaling and TCR stimulation *in vitro* is unstable. These signals induce transcription factors, such as NFAT, AP-1, and Smad3, which are capable of activating *Foxp3* transcription, and TGF-β signaling can also alter histone modifications of the *Foxp3* locus (Tone et al., 2008). However, these features cannot be maintained once TGF-β signaling and TCR stimulation are lost, resulting in loss of *Foxp3* transcription (Ohkura et al., 2012).

One of the consequences of having Treg-specific DNA demethylation is enhanced and ensured expression of Treg signature molecules by increasing accessibility of enhancers by constitutively expressed transcription factors. In general, DNA methylation interferes with binding of transcriptional factors by masking the consensus sequence with methyl group or by preferentially attracting methyl-CpG-binding proteins such as MBD family members, MeCP2 and Kaiso (Tost, 2010). Thus, removal of methyl group from DNA increases the accessibility for transcriptional factors and allows their transcriptional regulation. In fact, insertion of non-methylated *Foxp3* CNS2 region, but not methylated one, into a reporter construct significantly increased the luciferase reporter activity (Schmidl et al., 2009; Polansky et al., 2010). This indicates that *Foxp3* CNS2 contains a transcriptional enhancer which is normally hidden by DNA methylation but becomes active along Treg cell development. In line with this, CREB and Ets-1, transcription factors essential for Treg function, bind to CNS2 of *Foxp3* depending on its methylation status (Kim and Leonard, 2007; Mouly et al., 2010; Polansky et al., 2010). Furthermore, the transcriptional activation via this enhancer can be achieved by factors not specifically expressed in Treg cells, as similar increase in transcription activity occurred in both Tconv cells and Jurkat cells (Schmidl et al., 2009; Polansky et al., 2010). This suggests that once Treg-specific demethylation is complete, the target gene expression is ensured by constitutively expressed regulatory proteins as long as the methylation status is maintained. This role of DNA methylation status is further supported by the phenotypes of CNS2-null Treg cells, which lose Foxp3 expression gradually as they divide, demonstrating the link between transcriptional control at CNS2 and Foxp3 expression stability (Zheng et al., 2010). Since Treg-specific demethylated regions are present at the core set of Treg signature genes, epigenetic changes during Treg cell development, represented by DNA methylation status, may allow the phenotypes to be inherited over numerous cell divisions, with stabilization of the lineage commitment (Figure 2).

Collectively, these findings suggest that Treg-specific DNA hypomethylation is induced simultaneously with Foxp3 induction during natural Treg cell development and that these two molecular events generate Treg-type gene expression synergistically in some cases and independently in others. In addition, recent analysis of DNase I hypersensitivity regions in Treg cells has demonstrated differential DNase I sensitivity in a small fraction of genes in Treg cells, when compared with naïve T cells; and these genes mostly overlap with those that are specifically demethylated in Treg cells (Ohkura et al., 2012; Samstein et al., 2012). Since both high sensitivity to DNase I and DNA demethylation indicate an open chromatin state and high accessibility of regulatory proteins, these two events may be linked, possibly as consequences of chromatin remodeling. The mechanisms of epigenetic events which take place during Treg cell development and the precise contribution of these changes to the generation and maintenance of Treg cell characteristics remain to be elucidated. Yet, a high correlation of Treg-specific demethylation pattern with long-term stability and the function of Treg cells suggests that the epigenetic pattern can be a reliable marker to be used together with Foxp3 expression for identifying those Treg cells which have completed their lineage commitment.

## Establishment of Treg Cell Lineage

**Figure 3:**
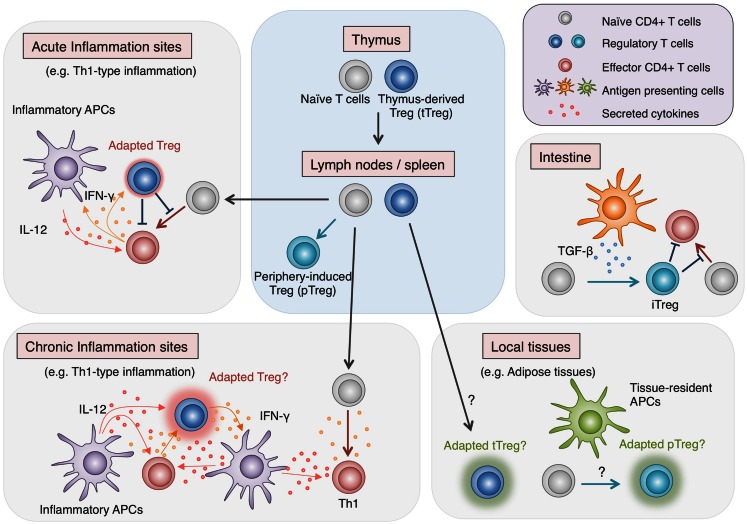
**Adaptability of Treg cells**. Treg cells effectively regulate immune responses in various contexts by flexibly adapting to the environments. While most Treg cells are generated in the thymus, some are induced from Tconv cells in the periphery, particularly in the intestine, where they play vital roles in maintaining the immune homeostasis with commensal microbes. Recent findings show that in local tissues such as adipose tissues, Treg cells, either induced locally or migrating from the lymphoid organs, exhibit unique characteristics, allowing specialized immune regulation (Cipolletta et al., 2012). Furthermore, during inflammation, Treg cells respond to environmental stimuli and adopt certain features of helper T cell characteristics to facilitate the immune regulation (Koch et al., 2009; Zheng et al., 2009). However, there are accumulating findings suggesting that strong stimulation by cytokines such as IL-12 induces not only the additional transcription factors and chemokine receptors but also pro-inflammatory cytokines in Treg cells (Oldenhove et al., 2009; McClymont et al., 2011; Zhao et al., 2011; Koenecke et al., 2012). Given the effects of pro-inflammatory cytokines in amplifying inflammation, possible cytokine production by Treg cells present potential hazard and might have relevance to chronic inflammation.

As discussed in this review, recent comprehensive analyses of Foxp3 protein complexes, genome-wide gene expression, and epigenetic modifications in Treg cells have revealed the complexity of molecular mechanisms responsible for generating Treg phenotypes. Since most of these characteristics are not controlled by Foxp3 alone, Treg cell development requires more than just the induction of Foxp3. The existence of non-suppressive Foxp3^+^ naïve-like non-Treg cells also shows the difficulty of reliable Treg delineation by Foxp3 expression alone. Treg cells which have undergone specific epigenetic programs show Treg-specific DNA demethylation as well as Foxp3 expression and exhibit full spectrum of Treg-type gene expression profile, indicating that epigenetic conversion, induction of the core set of transcription factors, formation of protein complexes are likely to occur simultaneously during the development of Treg cells. Notably, active DNA demethylation at *Foxp3* CNS2 region takes place during the thymic Treg cell development, in parallel with the induction of Treg-type gene expression and is completed as Treg cells migrate to the periphery (Toker et al., 2013). Treg-specific DNA hypomethylation is similarly observed in periphery-induced pTreg cells and there is no significant difference in gene expression, with some exceptions, between tTreg cells and pTreg cells (Haribhai et al., 2011; Ohkura et al., 2012). Recent studies have identified subpopulations of Treg cells with distinct expression of additional transcription factors such as T-bet, IRF4, Bcl6, and PPARγ (Koch et al., 2009; Zheng et al., 2009; Linterman et al., 2011; Cipolletta et al., 2012). Although the increase in phenotypic diversity within such Treg cell populations apparently indicates the existence of heterogeneous Treg subtypes, it may merely demonstrate the flexibility of natural Treg cells, adapting to each immunological context for effective immune suppression (Figure 3). As illustrated by the difference between Treg cells and Foxp3^+^ naïve-like non-Treg cells, protein expression can be transient and unstable, yet once the epigenetic regulation is established to ensure the stability of key regulator expression, the cells may achieve their lineage commitment and maintain the phenotypes in various immunological contexts.

**Figure 4:**
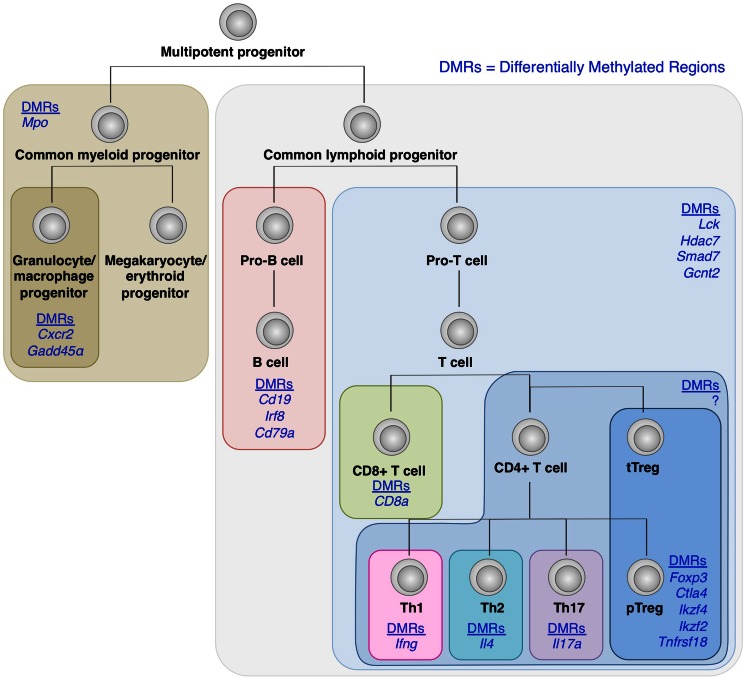
**DNA demethylation during hematopoietic cell differentiation**. Differentially methylated regions (DMRs) tends to be detected within genes encoding molecules associated with lineage specification, such as *Cxcr2* and *Gadd45*α in granulocyte/macrophage progenitors; *Cd19*, *Irf8*, and *Cd79*α in B cell lineage; and *CD8a* in CD8^+^ T cells (Ji et al., 2010; Bock et al., 2012; Lee et al., 2012). Similarly, within CD4^+^ T cell subsets, lineage-specific DNA demethylation occurs within genes encoding molecules involved in cell subset-specific functions (Lee et al., 2002; Ansel et al., 2003; Wilson et al., 2009; Cohen et al., 2011; Ohkura et al., 2012; Thomas et al., 2012). These findings suggest the involvement of epigenetic regulations during cell fate determination and linage commitment.

Recent genome-wide mapping of DNA methylation status in a number of hematopoietic cells has revealed that as hematopoietic stem cells undergo differentiation into different lineages such as T cell and B cells, and CD4^+^ and CD8^+^ T cells, lineage-specific genes are increasingly demethylated, whereas genes associated with other lineages become methylated, in cells committed to a particular cell lineage (Ji et al., 2010; Bock et al., 2012; Lee et al., 2012) (Figure 4). Naïve T cells can also differentiate into helper T (Th) cells such as Th1, Th2, Th17, Tfh, and presumably Th9, Th22 cells in the periphery depending on environmental stimuli. Key transcription factors such as T-bet, GATA-3, and RORγt, which modulates a large set of gene expression to specify the phenotypes and functions of Th1, Th2, and Th17 subset, respectively. Being similar to the case with Foxp3 and Treg development, Th cell differentiation is likely to involve epigenetic conversion in addition to the induction of transcription factors. Indeed, like Treg cells, which show a specific DNA methylation pattern distinct from naïve T cells, these Th cell subsets possess specific DNA methylation patterns of the genes encoding cytokines and transcription factors associated with each subset (Lee et al., 2002; Ansel et al., 2003; Wilson et al., 2009; Cohen et al., 2011; Ohkura et al., 2012; Thomas et al., 2012). Furthermore, exposing Th1 cells to Th17-inducing stimuli results in altered gene expression accompanying histone modification, but not DNA demethylation of Th17-specific genes such as the *Il17a* gene; similarly, Th17 cells in Th1-polarizing conditions do not acquire DNA demethylation of Th1-specific genes such as the *Ifng* gene (Cohen et al., 2011).

Taking these findings together, it is likely that changes in environmental stimuli, for example, due to different types of inflammation, may temporarily alter the gene expression and histone modification, and render highly differentiated Treg or Th cells adaptive to the environment with apparent plasticity, yet their DNA methylation status may determine their basic cell lineage commitment. However, assuming that even epigenetic changes are theoretically reversible, plasticity of differentiated cells needs further investigation to clarify whether any stimulation is able to change DNA methylation status of terminally differentiated cells, such as Treg cells, and drive them differentiate into other lineages.

## Are Treg Cells Plastic?

A number of recent reports have demonstrated possible plasticity of Treg cells. Under a physiological condition, a fraction of murine CD4^+^Foxp3^+^ T cells appear to lose Foxp3 expression and become exFoxp3 T cells (Komatsu et al., 2009; Zhou et al., 2009). Furthermore, the conversion of Foxp3^+^ T cells to exFoxp3 T cells is enhanced under lymphopenic conditions and Th1- and Th17-polarizing conditions both *in vivo* and *in vitro* (Xu et al., 2007; Yang et al., 2008; Oldenhove et al., 2009; Yurchenko et al., 2012). Similarly, human Foxp3^+^ T cells also contain a fraction with unstable Foxp3 expression (d’Hennezel et al., 2011). In contrast, another study has demonstrated that Treg cells in peripheral lymphoid organs are capable of stably maintaining Foxp3 expression *in vivo* even under inflammatory conditions (Rubtsov et al., 2010). Analysis of DNA methylation status of the *Foxp3* gene shows that the Treg plasticity can simply be attributed to the presence of a minor fraction of Foxp3^+^ T cells which lack *Foxp3* hypomethylation (Miyao et al., 2012). Therefore, controversy regarding Treg plasticity may be partly due to experimental variables; particularly in lymphopenic and IL-2 deficient conditions, expansion of Foxp3^+^ naïve-like non-Treg cells and apoptosis of stable Treg cells may appear as dramatic loss of Foxp3 expression in Treg cells. As discussed in this review, Foxp3^+^ T cells include Treg cells and non-Treg cells and it should be determined whether current phenomena are due to the instability of the latter, or both. It is important to resolve this matter of Treg plasticity, since some of these exFoxp3 T cells possess auto-reactive TCRs, and thus possibility of becoming harmful autoimmune effector T cells with the capacity to secrete pro-inflammatory cytokines (Zhou et al., 2009).

This plasticity issue also raises questions regarding the concept of lineage commitment in T cell subsets. Is there a clear borderline between each subset? If there are distinct signals to convert naïve T cells into each T helper or Treg cell lineage, what happens when Treg cells receive stimulation for T helper cell specification? Is there a mechanism to prevent reprograming once the Treg lineage is established? On this matter, the relationship between Treg cells and Th1 cells are well demonstrated by Koch et al. Treg cells express T-bet and CXCR3 upon exposure to IFN-γ; however, further progression into Th1 differentiation is aborted since Treg cells show delayed expression of IL-12 receptor, therefore being less responsive to IL-12 signaling, which is required for IFN-γ production (Koch et al., 2012). However, this scenario may only apply to acute Th1-type infection where IL-12 production is transient enough to limit the IL-12 receptor expression on T-bet^+^ Treg cells. Other reports have demonstrated the ability of Treg cells to produce IFN-γ; for example, IFN-γ-producing Foxp3^+^ Treg cells are observed *in vivo* during viral infections and acute graft-versus-host disease in mice, and in patients with multiple sclerosis and type I diabetes mellitus, and in several *in vitro* studies (Oldenhove et al., 2009; Dominguez-Villar et al., 2011; McClymont et al., 2011; Zhao et al., 2011; Koch et al., 2012; Koenecke et al., 2012). If cytokines are capable of reprogramming Treg cells to produce pro-inflammatory cytokines, it is potentially dangerous as it could amplify the inflammatory responses by converting Treg cells to act like effector T cells during chronic inflammation. It is noted, however, that the assessment of cytokine secretion often involves prior stimulation with PMA/Ionomycin and whether Treg cells actually produce significant amount of pro-inflammatory cytokines *in vivo* is unclear. Future studies need to address whether these scenarios are relevant during human diseases, whether there are alternative failsafe mechanisms to prevent the reprograming of Treg cells or whether this is the limit of Treg stability.

## Future Perspectives for Clinical Application of Treg Cells

In this review, we have discussed how Treg cells can be molecularly defined as a cellular entity. Differences among functional definition (CD4^+^ T cells with suppressive function), molecular definition (CD4^+^Foxp3^+^ T cells), and epigenetic definition (cells with Treg-specific DNA methylation status) of Treg cells are negligible in most physiological settings. However, in the contexts of various immunological diseases, the accuracy of Treg definition matters. Some of the human naïve T cells and effector T cells are capable of expressing Foxp3 in response to TCR activation (Allan et al., 2007). In chronic autoimmune diseases, Foxp3 may be easily expressed in activated Tconv cells by frequent TCR stimulation and this could potentially mask the underlying Treg deficiency and/or dysfunction. Among currently identified autoimmune disorders, only few of them are clearly linked to Treg abnormality despite the well-studied roles of Treg cells in the maintenance of self-tolerance (Gregersen and Behrens, 2006; Buckner, 2010). This is partly due to technical difficulty to precisely assess Treg dysfunction, particularly if Foxp3^+^ T cells are present at a normal or increased frequency. With the epigenetic features of Treg cells revealed, it may be used as a new tool for assessing Treg function and for better understanding of disease pathology.

Treg cells have crucial roles in maintaining immunological self-tolerance and homeostasis and are suspected to be involved in a variety of immunological disorders (Shevach, 2000; Maloy and Powrie, 2001; Sakaguchi, 2004). Treg cells thus possess the potential to fix a wide range of immunological diseases from allergy to cancer. For treatment of autoimmune disorders and allergy and for efficient acceptance of grafts after transplantation, adoptive transfer of Treg cells expanded *ex vivo* or induced *in vitro* is promising. The ultimate goal of this approach is to control inflammation with minimum adverse effects by using antigen-specific Treg cells. However, little progress has been made toward practical application of this idea due to the plasticity of some Foxp3^+^ T cells and the lack of reliable cell surface markers for differentiating human Treg cells from other activated T cells, which would increase the chances of non-Treg cells or unstable Treg cells being contaminated and thus raise the concerns regarding safety and efficacy of Treg cell therapy (Riley et al., 2009). Given low frequency of Treg cells in human peripheral blood, an ideal approach is to generate stable antigen-specific Treg cells *in vitro* from Tconv cells. Yet, current method of iTreg generation using TGF-β and IL-2 can induce Foxp3 protein expression, but these iTreg cells are significantly different from *in vivo* Treg cells in terms of gene expression, epigenetics, stability, and function (Ohkura et al., 2012). As discussed in this review, the epigenetic conversion and Foxp3 induction are critical determinants of generating and maintaining stable Treg cell lineage. The aims of future studies thus include better understanding of signals and mechanisms required for these two molecular events in the course of Treg cell development, which may help us identify ways to generate and expand stable Treg cells for therapeutic use.

## Conclusion

Treg cell development involves concurrent induction of Foxp3 expression and epigenetic conversion, which cooperatively generate Treg-type gene expression. It is possible to induce the expression of Foxp3 *in vitro*; however, often it is not accompanied by epigenetic changes or Treg-type gene expression. Foxp3 requires its co-factors to potentiate its function in gene regulation and confer suppressive activity on Treg cells. Furthermore, for long-term lineage commitment of Treg cells, the stability of Foxp3 and other Treg signature molecules need to be ensured by epigenetic modification represented by DNA methylation status. Further investigation will elucidate the mechanisms of epigenetic changes as well as Foxp3 induction in the course of Treg cell development, enabling us to devise new approaches for clinical application of Treg cells.

## Conflict of Interest Statement

The authors declare that the research was conducted in the absence of any commercial or financial relationships that could be construed as a potential conflict of interest.
